# Spontaneous hip ankylosis in end-stage osteoarthritis: a rare case of complete bony fusion

**DOI:** 10.11604/pamj.2025.50.36.46147

**Published:** 2025-01-29

**Authors:** Nikita Gangwani, Gurjeet Kaur

**Affiliations:** 1 Department of Musculoskeletal Physiotherapy, Ravi Nair Physiotherapy College, Datta Meghe Institute of Higher Education and Research (DU), Sawangi, Meghe, Maharashtra, Wardha, India,; 2 Ravi Nair Physiotherapy College, Datta Meghe Institute of Higher Education and Research (DU), Sawangi, Meghe, Maharashtra, Wardha, India

**Keywords:** Hip ankylosis, osteoarthritis, hip, spontaneous joint fusion

## Image in medicine

This case highlights a rare progression of hip osteoarthritis to spontaneous ankylosis, marked by unique clinical features. A 53-year-old male farmer presented with severe functional impairment due to complete loss of motion in his right hip, which progressed to painless ankylosis. Remarkably, the ankylosis occurred unilaterally without preceding trauma, inflammatory arthritis, or family history of joint disorders, contrasting with a well-functioning total hip replacement on the left side. Radiographs revealed total bony fusion of the right femoral head and acetabulum, with evident sclerosis and remodeling, while surrounding bone mineralization remained intact. The left hip arthroplasty components were well-positioned with no complications. This patient´s history, including a 50-pack-year smoking history and physically demanding occupation, likely contributed to accelerated joint degeneration and ankylosis. Muscle power around the right hip was graded at 2/5, with a 0° range of motion and notable iliopsoas and adductor tightness. Differential diagnoses such as post-traumatic ankylosis and ankylosing spondylitis were ruled out due to the absence of inflammatory markers or injury. This case is distinct as spontaneous ankylosis is a rare AVN outcome, particularly without surgical intervention. It underscores the need for early medical management of osteoarthritis and highlights the role of lifestyle and occupation in joint pathology. Its scientific significance lies in demonstrating the complex mechanisms of joint degeneration and the importance of personalized approaches to joint health.

**Figure 1:**
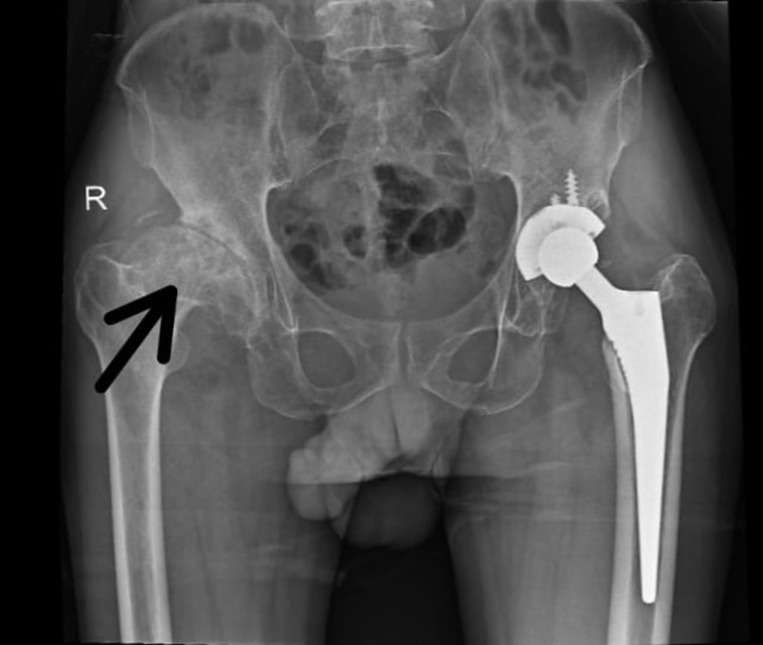
the right hip shows complete loss of joint space with bony fusion between the femoral head and acetabulum, consistent with spontaneous ankylosis, sclerosis, and bone remodeling around the ankylosed joint; the left hip demonstrates well-positioned total hip arthroplasty components without evidence of loosening or hardware complications, and the remaining pelvic bones appear intact with normal mineralization

